# Prevention and Management of Gastroesophageal Varices in Cirrhosis

**DOI:** 10.1155/2012/750150

**Published:** 2012-04-22

**Authors:** Yen-I Chen, Peter Ghali

**Affiliations:** ^1^Division of Hepatology and Gastroenterology, McGill University Health Center, McGill University, Montreal, QC, Canada H3A 1A1; ^2^Internal Medicine Office, Jewish General Hospital, Montreal, QC, Canada H3T 1E2

## Abstract

Variceal hemorrhage is one of the major complications of liver cirrhosis associated with significant mortality and morbidity. Its management has evolved over the past decade and has substantially reduced the rate of first and recurrent bleeding while decreasing mortality. In general, treatment of esophageal varices can be divided into three categories: primary prophylaxis (prevention of first episode of bleeding), management of acute bleeding, and secondary prophylaxis (prevention of recurrent hemorrhage). The goal of this paper is to describe the current evidence behind the management of esophageal varices. We will discuss indications for primary prophylaxis and the different modes of therapy, pharmacological and interventional treatment in acute bleeding, and therapeutic options in preventing recurrent bleeding. The indications for TIPS will also be reviewed including its possible benefits in acute variceal hemorrhage.

## 1. Introduction

Portal hypertension in liver cirrhosis results from the anatomical changes and the development of contractile element in the liver vascular bed secondary to progressive hepatic fibrosis and formation of regenerative nodules [1, 2]. The increase in portal pressure triggers splanchnic vasodilation, increased cardiac output, and fluid/salt retention leading to a hyperdynamic circulation and increased portal flow. Formation of collaterals between the portal and systemic systems such as those found in the lower esophagus and gastric cardia (gastroesophageal varices) may not only relieve some of the pressure, but also pose a risk for rupture and bleeding [2].

The prevalence of gastroesophageal varices ranges from 0–40% in compensated cirrhosis to 70–80% in decompensated liver disease, while their growth and progression occur at an estimated 7% per year [2, 3]. The one-year rate of first variceal hemorrhage is 5% for small varices and 15% for large varices [4]. Advanced liver disease (Child B or C), large varices, and varices with red wale marks are bad prognostic signs associated with higher incidence of bleeding. Six-week mortality with each episode of bleeding varies between 15 and 20% and is largely dependent on the severity of the liver disease (0% for Child A and 30% for Child C) [5–7]. Finally, the 1-year rebleeding rate following initial variceal hemorrhage is approximately 60% [8].

The current treatment of gastroesophageal varices has substantially reduced the rate of first and recurrent bleeding while decreasing the mortality of acute variceal hemorrhage [9, 10]. The purpose of this paper is to summarize the management of gastroesophageal varices in terms of primary prophylaxis (prevention of first episode of bleeding), treatment of acute hemorrhage, and secondary prophylaxis (prevention of recurrent bleeding).

## 2. Primary Prophylaxis

Nonselective *beta*-blockers are the current mainstay of therapy in the prevention of first episode variceal hemorrhage [9–11]. *β*1 inhibition reduces cardiac output while *β*2-blockade induces splanchnic vasoconstriction and together it results in decreased portal flow and pressure [12]. Nonselective *beta*-blockers used for primary prophylaxis in North America include propranolol and nadolol. Carvedilol has recently been investigated in portal hypertension given its *alpha*-blocking component and its potential to better diminish portal pressure [13]. However, more data will be needed on its effectiveness and long-term safety. At this time it is premature to endorse Carvedilol as a first-line agent for primary prophylaxis. In terms of dosing and goal of treatment, it is recommended to start at a low dose and to titrate up as tolerated until a heart rate of 55 beats/minute is achieved [9–11].

### 2.1. Cirrhosis with Small Varices

In patients with low-risk small varices (Child A without red wale marks), the use of nonselective *beta*-blocker is optional [9]. There is limited evidence showing that nonselective *beta*-blockers may slow the growth of varices but they do not reduce mortality and their use cannot be universally recommended over regular endoscopic surveillance (every 2 years and annually with hepatic decompensation) [14]. However, nonselective *beta*-blockers are recommended in patients with small varices and high-risk features such as red wale marks and/or Child B-C cirrhosis [9].

### 2.2. Cirrhosis with Medium to Large Varices

A large meta-analysis looking at propranolol/nadolol versus placebo, in patients with cirrhosis and medium to large varices, found a significantly lower incidence of first variceal bleeding in the treatment group: 14% compared to 30% [4]. Also, these nonselective *beta*-blockers may be equivalent to endoscopic variceal band ligation (EVBL) in terms of primary prevention and mortality rate [15, 16]. In addition, they are inexpensive and can potentially prevent other complications of cirrhosis such as spontaneous bacterial peritonitis and bleeding from portal hypertensive gastropathy [17, 18]. However, 15–20% of patients treated with nonselective *beta*-blockers are noncompliant due to common side effects such as fatigue, dizziness, and shortness of breath. EVBL is associated with fewer side effects and does not rely on patient compliance but requires technical expertise and can lead to serious complications such as bleeding from ligation-induced ulcers [12, 19]. Finally, a randomized controlled trial comparing EVBL and propranolol to EVBL alone in patients with large varices did not show a difference in terms of mortality or incidence of first bleed [20]. Therefore, depending on patient/physician preference and available expertise either nonselective *beta*-blocker or EVBL alone should be used for primary prophylaxis in cirrhosis with medium to large varices. Combination therapy does not seem to confer any additional benefit (Table 1).

## 3. Acute Variceal Bleeding

The management of acute variceal bleeding with the combination of vasoconstrictors, endoscopic therapy, and antibiotics has decreased mortality substantially [9, 10]. Initial assessment of a patient with acute variceal hemorrhage begins with the evaluation of airway, breathing, and circulation. Many of these patients are at risk for aspiration and intubation is often performed for airway protection, although there are limited data to justify this practice [21, 22]. Volume resuscitation with blood and fluid is essential in the initial stabilization; however, experimental studies suggest that overly aggressive volume repletion can worsen bleeding and increase the rate of rebleeding and mortality [23]. Therefore, meticulous resuscitation with a target hemoglobin level of 8 g/dL is recommended [23, 24]. In addition, animal studies suggest that blood transfusion may be superior to fluid administration given that fluid resuscitation may decrease blood viscosity, which can exacerbate portal pressure and potentially worsen acute variceal hemorrhage [25]. Correction of significant coagulopathy and thrombocytopenia with fresh frozen plasma and platelet transfusions should also be considered [10]. Studies on Factor VIIa have failed to show benefit in terms of mortality and control of bleeding and its use is currently not recommended [6].

## 4. Antibiotics in Acute Variceal Bleeding

Acute variceal hemorrhage has been shown to increase the risk for severe bacteremia, which is associated with higher mortality rates and greater incidence of rebleeding [26–28]. Meta-analyses have revealed that antibiotic prophylaxis can improve short-term survival while decreasing bacterial infections and rebleeding rates [26, 29, 30]. Oral norfloxacin or intravenous ciprofloxacin for 7-days, administered at the time of acute bleeding, works by decreasing the amount of gram-negative bacteria in the gut believed to be the most common source for infection in cirrhosis [27, 28]. However, in patients with advanced cirrhosis (Child B/C) ceftriaxone may be superior to norfloxacin in preventing infections [28]. This is likely due to ceftriaxone's extended coverage against nonenterococcal streptococci and quinolone-resistant Gram-negative bacteria. Therefore, cirrhotic patients without advanced liver insufficiency and acute variceal hemorrhage should receive either oral norfloxacin or IV ciprofloxacin for 7 days while ceftriaxone is preferred in patients with Child B/C cirrhosis or previous quinolone use.

## 5. Pharmacological Therapy and Endoscopic Management

In addition to antibiotics, vasoactive agents such as vasopressin, terlipressin, somatostatin, and octreotide play a major role in controlling acute esophageal variceal hemorrhage through their ability to induce splanchnic vasoconstriction thereby reducing portal flow and pressure [9, 10]. In fact, they may be equally as effective as endoscopic sclerotherapy in controlling initial bleeding and in preventing rebleeding with less adverse effects [31, 32]. These agents, when administered at the time when variceal bleeding is suspected, can achieve initial hemostasis in 60–80% of the cases [33].

Vasopressin is a potent vasoconstrictor that reduces blood flow to all splanchnic organs leading to substantial decreases in portal pressure [10]. However, its use has been limited by its side effects such as hypertension, cardiac, and peripheral ischemia, and ischemic bowel. Terlipressin is a synthetic analogue of vasopressin with longer pharmacological activity and fewer side effects [4, 10, 34]. The intact molecule has immediate vasoconstrictive activity, which is followed by a delayed effect secondary to a slow enzymatic breakdown of terlipressin into vasopressin. It is the only agent that has been demonstrated to decrease mortality in acute variceal hemorrhage [4]; however, it is not yet available in North America. In terms of dosing, terlipressin is given intravenously and should be started at 2 mg every 4 hours for 48 hours, followed by 1 mg every 4 hours [9]. The optimal duration of treatment is unknown but current recommendations suggest a total of 2–5 days.

Somatostatin is a naturally occurring tetradecapeptide that has inhibitory effects on exocrine/endocrine hormones, gastrointestinal motility, and systemic blood flow leading to a decreased circulation and pressure in the portal and porto-collateral system [34]. Octreotide, a synthetic analogue of somatostatin with greater potency and longer half-life, is the only substance available in North America mainly due to its safety profile and its apparent effectiveness when used in combination with EVBL [9, 34, 35]. However, its effectiveness in controlling acute variceal hemorrhage has not been firmly established [11, 34]. In terms of dosing, it is administered intravenously and should be initiated with a 50 mcg bolus followed by an infusion at 50 mcg/hr [9]. A bolus can be repeated in the first hour if variceal hemorrhage is uncontrolled. As with terlipressin therapy should be continued for 2–5 days (Table 2).

Following initiation of vasoactive agents, EGD should be performed within 12 hours of presentation [10]. EVBL is superior to sclerotherapy and is the modality of choice [19]. The combination of EVBL with pharmacological therapy is the current standard of care and when compared to EVBL alone it improves initial control of bleeding and 5-day hemostasis without a mortality benefit [36].

## 6. Role of TIPS in Acute Variceal Bleeding

The use of transjugular intrahepatic portosystemic shunt (TIPS) in acute variceal hemorrhage has been historically reserved for salvage therapy in patients who have failed endoscopic and pharmacological treatment. However, a recent randomized controlled trial looking at early TIPS, defined as within 72 hours of standard therapy (EVBL + antibiotic + vasoactive agent), versus standard therapy alone showed that in patients with Child B/C cirrhosis the early use of TIPS was associated with a reduction in the failure to control bleeding, lower incidence of rebleeding, and decreased mortality rate [37]. In addition, the TIPS group did not have an increased incidence of hepatic encephalopathy. Notably, however, the outcomes in the nonearly TIPS group were unusually poor. Although more studies will be needed to confirm these findings, the early use of TIPS should be considered in patients with severe liver disease who present with acute variceal bleeding following initial standard therapy.

## 7. Secondary Prevention

Patients who survive an episode of acute variceal hemorrhage are at high risk of recurrence. Overall, 60% of these individuals will rebleed within 2 years with a mortality rate of 33% [4, 8]. Therapy aimed at reducing this risk is essential and should be implemented as soon as the initial hemorrhage is controlled [9, 10]. Multiple modes of treatment have been investigated including monotherapy with nonselective *beta*-blockers, combination medical therapy, EVBL with or without pharmacological adjunct, and TIPS.

Nonselective *beta*-blockers have been shown to decrease rebleeding rates from 60% to 42-43% likely secondary to the decrease in portal pressure [4, 8, 24]. Further portal pressure reduction can be achieved when they are combined with oral nitrates (ISMN) [38]. Nitrate-induced venodilation decreases cardiac output and blood pressure, which can lead to a baroreceptor-mediated splanchnic vasoconstriction and fall in portal pressure [39]. It may also have a direct vasodilatory effect on the portosystemic circulation; however, a randomized trial and a recent meta-analysis did not show any benefit in adding a nitrate. In addition, combined therapy is associated with more adverse effects leading to discontinuation of treatment [40, 41].

In terms of endoscopic therapy, EVBL is superior to sclerotherapy and is the method of choice [42, 43]. Meta-analysis of several randomized controlled trials (719 patients) comparing EVBL versus combination medical therapy, with nonselective *beta*-blockers and nitrates, showed no difference in rebleeding rate and increased survival in the medically treated group [44–47]. Also, two prospective trials suggest that the combination of EVBL with medical therapy (nadolol) may be superior to EVBL alone [48, 49]. The use of EVBL and a nonselective *beta*-blocker is the current recommendation for secondary prophylaxis and should be instituted without delay following initial bleed [10]. However, a recent randomized controlled trial looking at combination therapy (EVBL + nadolol + nitrate) versus medical therapy alone (nadolol + nitrate) found no difference in rebleeding rates, need for rescue therapy, or mortality while the combination therapy was associated with more adverse events [50]. More studies will be needed to confirm these findings but future guidelines may move towards medical therapy alone.

Finally, TIPS in secondary prophylaxis has been shown to lower rebleeding rates when compared to the aforementioned medical/endoscopic therapy [51–53]. However, no mortality benefit has been demonstrated with TIPS and its use is associated with higher costs and incidence of hepatic encephalopathy. Therefore, the use of TIPS in secondary prophylaxis is not recommended; however, its use may be considered following failure with conventional medical therapy [10]. This may change with the advent of polytetrafluoroethylene- (PTFE-) covered prostheses, which substantially improves TIPS patency.

In summary, EVL in combination with nonselective *beta*-blockers is the method of choice in preventing recurrent variceal bleeding. The addition of nitrates can theoretically potentiate the portal pressure drop; however, it has not been shown to decrease mortality or rebleeding rates and is associated with greater side effects. TIPS is not recommended for secondary prophylaxis and should only be considered following failure with usual medical therapy. It decreases rebleeding rates without a mortality benefit while being associated with higher costs and incidence of hepatic encephalopathy. Whether the new PTFE covered stent will improve TIPS efficacy in secondary prophylaxis remains to be seen, but for the moment its use is restricted to those cases where other therapies have failed.

## 8. Conclusion

The management of gastroesophageal varices has evolved over the last decade resulting in improved mortality and morbidity rates. Primary prevention with nonselective *beta*-blockers or EVBL should be initiated in all patients with medium to large varices and in patients with small varices associated with high risk features such as red wale marks and/or advanced cirrhosis. While prophylaxis in patients with small varices without high risk features is considered optional. In acute bleeding, vasoactive agents such as octreotide or terlipressin should be initiated along with antibiotics followed by EVBL within 12 hours of presentation. These patients are at increase risk for rebleeding and secondary prevention should be initiated immediately following control of initial hemorrhage with serial EVBL and nonselective *beta*-blocker. Currently, TIPS' role in secondary prophylaxis is limited except for failure with conventional therapy; however, this may change with the advent of PTFE covered stents. Although therapy for patients with varices has made significant progress, it will continue to improve with better endoscopic technique, novel pharmacological agents, greater efficiency of liver transplant, and more effective rescue therapy.

## Figures and Tables

**Table 1:** Primary prophylaxis and surveillance.

Surveillance/prophylaxis modalities	Indications	Dose	Goal
Endoscopic surveillance	Low-risk* small varices (not on nonselective BB**)	Every 2 years and annually with liver decompensation	Surveillance for progression into higher-risk lesions needing medical or endoscopic prophylaxis
Nadolol	High-risk*** small varices and medium-large varices *Optional*: low-risk small varices	Start: 40 mg qd	Titrate to heart rate: 55 beats/minute or maximally tolerated dose
Propranolol	High-risk small varices and medium-large varices *Optional*: low-risk small varices	Start: 10 mg bid	Titrate to heart rate: 55 beats/minute or maximally tolerated dose
EVBL	Medium to large varices	Every 2–4 weeks	Until variceal obliteration

*Low-risk: Child A cirrhosis and no red wale marks, ** beta-blocker, ***high-risk: Child B or C cirrhosis and/or presence of red wale marks.

**Table 2:** Initial medical management of acute variceal bleeding.

Treatment	Dose	Duration	Details
Antibiotics			
Ceftriaxone	1 g IV daily	5–7 days	Severe cirrhosis Child B/C and/or high suspicion of quinolone resistance
Ciprofloxacin	400 mg IV or 500 mg oral twice daily	5–7 days	Mild cirrhosis Child A and low suspicion of quinolone resistance
Norfloxacin	400 mg oral twice daily	5–7days	Mild cirrhosis Child A and low suspicion of quinolone resistance
Vasoconstrictors			
Octreotide	50 *μ*g IV bolus, then infusion at 50 *μ*g/hr	2–5 days	Initial bolus can be repeated in the first hour if bleed not controlled
Terlipressin	2 mg IV every 4 hr × 48 hr, then 1 mg IV every 4 hr	2–5 days	Not available in North America
Somatostatin	250 *μ*g IV bolus, then 250–500 *μ*g/hr	2–5 days	Not available in North America
